# In-Hospital Mortality Outcomes of ST-Segment Elevation Myocardial Infarction: A Cross-Sectional Study from a Tertiary Academic Hospital in Johannesburg, South Africa

**DOI:** 10.3390/jcdd10080348

**Published:** 2023-08-15

**Authors:** Lindokuhle Ndaba, Arthur Mutyaba, Dineo Mpanya, Nqoba Tsabedze

**Affiliations:** Division of Cardiology, Department of Internal Medicine, School of Clinical Medicine, Faculty of Health Sciences, University of the Witwatersrand, Johannesburg 2193, South Africa; 533584@students.wits.ac.za (L.N.); arthur.mutyaba@wits.ac.za (A.M.); dineo.mpanya@wits.ac.za (D.M.)

**Keywords:** acute coronary syndrome, cardiovascular epidemiology, mortality, percutaneous coronary intervention, ST-segment elevation myocardial infarction, Sub-Saharan Africa

## Abstract

In sub-Saharan Africa, the burden of atherosclerotic cardiovascular disease (ASCVD) is increasing. This study aimed to describe the clinical characteristics of patients with ST-segment elevation myocardial infarction (STEMI) and estimate the in-hospital all-cause mortality rate. We conducted a cross-sectional retrospective single-centre study of STEMI patients who underwent diagnostic coronary angiography with or without percutaneous coronary intervention (PCI) between January 2015 and December 2019. We compared demographic and clinical parameters between survivors and non-survivors with descriptive statistics. Univariable and multivariable logistic regression analyses were performed to determine the predictors of all-cause mortality. The study population consisted of 677 patients with a mean age of 55.5 ± 11.3 years. The in-hospital all-cause mortality rate was 6.2% [95% confidence interval (CI): 4.5–8.3%]. Risk factors for ASCVD included smoking (56.1%), hypertension (52.8%), dyslipidemia (40.0%), and a family history of coronary artery disease (32.7%). A pharmaco-invasive management strategy (treatment with thrombolytic therapy and PCI) was implemented in 36.5% of patients and reduced all-cause mortality risk (OR: 0.16; CI: 0.04–0.71, *p* = 0.015). The in-hospital all-cause mortality rate in STEMI patients was 6.2%, and a pharmaco-invasive management strategy proved to be an effective approach.

## 1. Introduction

In high-income countries (HICs), there has been a notable decrease in the mortality rate associated with ST-segment elevation myocardial infarction (STEMI) [1]. However, sub-Saharan Africa (SSA) has experienced a significant surge in risk factors related to STEMI, such as hypertension, smoking, dyslipidemia, and diabetes mellitus. The increase in the prevalence of risk factors for atherosclerotic cardiovascular diseases (ASCVD) can be attributed to urbanisation, dietary and lifestyle changes, and an ageing population [2,3]. Consequently, ischemic heart disease (IHD), cerebrovascular disease, and hypertensive heart disease have emerged as the leading causes of cardiovascular mortality in SSA [3]. The increasing and high burden of non-communicable diseases in SSA has put immense strain on an already overwhelmed and under-equipped healthcare infrastructure.

In SSA, the mortality rate associated with STEMI exhibits considerable variation and can be as high as 24.5% [2]. Timely access to coronary revascularisation strategies is a critical determinant of mortality outcomes in SSA among patients with acute coronary syndromes (ACS) [2]. However, several factors contribute to the mortality burden, including lack of patient education, delayed health-seeking behaviour, unequal healthcare systems, inappropriate triage, incomplete revascularisation, and limited access to primary percutaneous coronary intervention (PCI) [2].

A review of the South African arm of the Acute Coronary Events (ACCESS) registry, a multinational survey of current management strategies of patients hospitalised with ACS in 19 African countries, likely underestimated the in-hospital mortality rate, due to a small representation of South African patients in the registry, with only 642 out of 12,068 patients, and 253 individuals with confirmed STEMI [4]. There are limited data reporting outcomes in patients with STEMI residing in SSA, particularly in South Africa. This study aimed to describe risk factors and the clinical presentation of patients hospitalised with STEMI in a tertiary academic hospital in Johannesburg, South Africa. Furthermore, we aimed to determine the in-hospital all-cause mortality rate and identify predictors of all-cause mortality among STEMI patients.

## 2. Materials and Methods

### 2.1. Study Design, Study Setting and Participants

We conducted a cross-sectional retrospective single-centre study of consecutive patients diagnosed with STEMI between 1 January 2015 and 31 December 2019. The study was conducted at the Charlotte Maxeke Johannesburg Academic Hospital (CMJAH) in Johannesburg, South Africa. The CMJAH is a state-owned academic tertiary hospital in central Johannesburg, forming part of the University of the Witwatersrand academic cluster of teaching hospitals. Patients 18 years and older who met the inclusion criteria for STEMI, per the third and fourth Universal Definitions of Myocardial Infarction, were included in the study [5,6]. An overlap in the definitions occurred because the third universal definition was released in 2012, while the fourth universal definition was released in 2018. Consequently, STEMI patients hospitalised between 2015 and 2018 were diagnosed with myocardial infarction (MI) using the third universal definition, whereas patients seen in 2018 and 2019 were diagnosed using the fourth universal definition.

Patients with incomplete medical records (pertinent missing data include gender, ACS presentation, electrocardiogram (ECG) findings, laboratory results, intervention, and clinical outcome), duplicate medical records, a working diagnosis of non-ST elevation acute coronary syndromes (NSTACS), and alternate diagnoses such as acute peri-myocarditis, takotsubo cardiomyopathy, and trauma-related myocardial infarction were excluded from the final analysis. Ethics approval was obtained from the University of the Witwatersrand Human Research Ethics Committee (Clearance Certificate Number: M201095) and relevant hospital authorities. The authors adhered to the Strengthening the Reporting of Observational Studies in Epidemiology (STROBE) guidelines [7].

### 2.2. Data Collection

**Figure 1:**
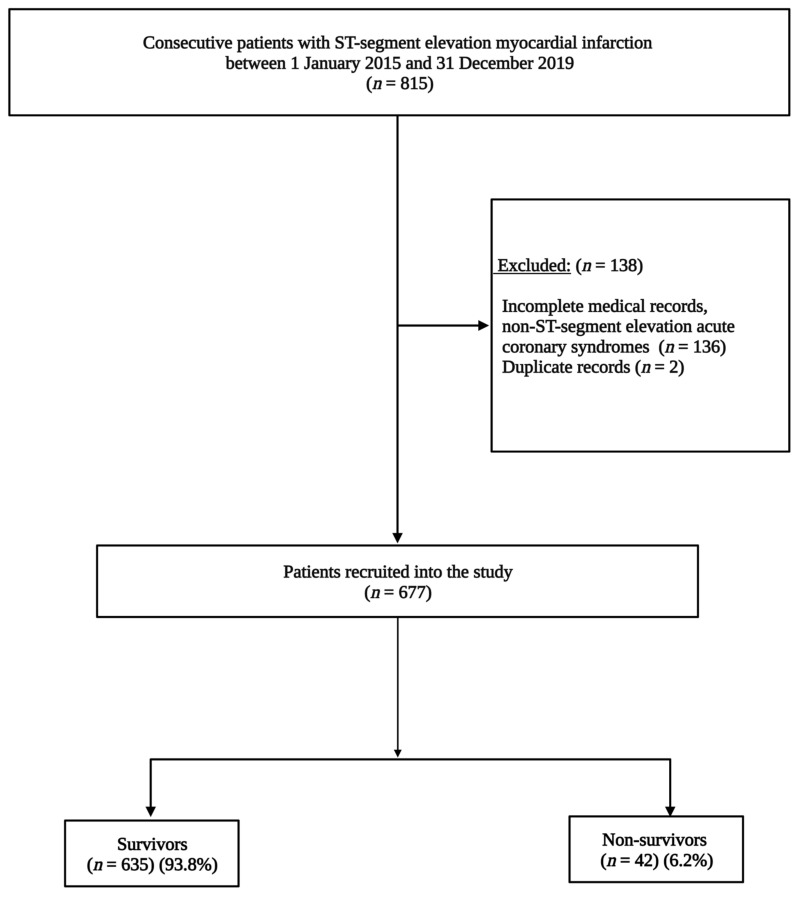
Flow chart outlining enrolment of patients into the study. STEMI: ST-segment elevation myocardial infarction.

Inpatient medical records for 815 patients were retrieved. A verification process entailed a review of the medical records, archived coronary angiogram reports, the coronary angiogram suite admission records, and the Cardiac Intensive Care Unit (CICU) admission records. After excluding patients with incomplete medical records, duplicate medical records, NSTACS, and alternative diagnoses, 677 STEMI patients were included in the final study analysis (Figure 1).

Data collected included demographics (age, gender, and ethnicity), co-morbidities, smoking, clinical presentation (onset of chest pain, time from onset of chest pain to first medical contact, and catheterisation), ECG parameters (rhythm, rate, ST-segment elevation and depression, complete heart block (CHB), and bundle branch blocks), clinical parameters (blood pressure, New York Heart Association functional class, and Killip class) biochemical parameters (cardiac biomarkers, renal function, lipogram, and haemoglobin), coronary angiography findings (dominant coronary vessel, distribution of atherosclerotic disease on angiography, and the number of lesions), coronary intervention (adjunct medical therapy received, thrombolytic therapy, and PCI), post-intervention complications (arrhythmias and haemodynamic instability), medications, and in-hospital all-cause mortality. Data were captured in the Research Electronic Data Capture (REDCap) database.

In our study, STEMI patients were routinely managed with either one of four management strategies: (a) primary thrombolysis, (b) the pharmaco-invasive approach, (c) primary or ad hoc PCI without prior thrombolysis, and (d) conservatively in those presenting with a fully evolved myocardial infarct. The pharmaco-invasive approach involves thrombolysis followed by routine angiography with PCI. Conservative therapy referred to patients who underwent coronary angiography without prior thrombolysis and had no PCI. Furthermore, there was a primary or ad hoc PCI group consisting of patients who did not receive primary thrombolysis but had primary PCI or delayed angiography with PCI [8]. Lastly, the primary thrombolysis cohort received thrombolytic therapy (Streptokinase or Altepase) and angiography but did not undergo PCI. These distinctions allowed for a comprehensive analysis of the different treatment strategies and their outcomes.

### 2.3. Statistical Analysis

Frequencies and percentages were used to summarise categorical variables. The mean and standard deviation (SD) were used to summarise normally distributed continuous data, and the median and interquartile ranges (IQR) were used for continuous variables with a non-normal distribution. We compared demographic and clinical parameters between survivors and non-survivors. For continuous variables, the Student’s *t*-test was used to compare means for normally distributed variables. The Wilcoxon rank-sum test was used to compare medians for data with a non-normal distribution. Pearson’s Chi-square test was used to compare categorical variables. Univariable and multivariable logistic regression analyses were used to determine the predictors of in-hospital all-cause mortality. The odds ratios in the multivariable model were adjusted for confounders such as age and gender. After conducting a Pearson’s Chi-square test, Student’s *t*-test or a Wilcoxon rank-sum test, variables with a *p*-value < 0.1 were considered for further exploration in the univariable logistic regression model. We further included all variables from the univariable logistic regression model with a *p*-value less than 0.05 in the multivariable logistic regression model. A detailed explanation of how the multivariable logistic regression model was created is attached as Supplementary Materials. We presented the crude and adjusted odds ratios with their 95% confidence intervals (95% CI). Statistical significance was considered at a *p*-value < 0.05. Analysis was performed using Stata version 17 (StataCorp Ltd., College Station, TX, USA).

## 3. Results

### 3.1. Demographic and Clinical Characteristics of Patients with ST-Segment Elevation Myocardial Infarction

**Figure 2:**
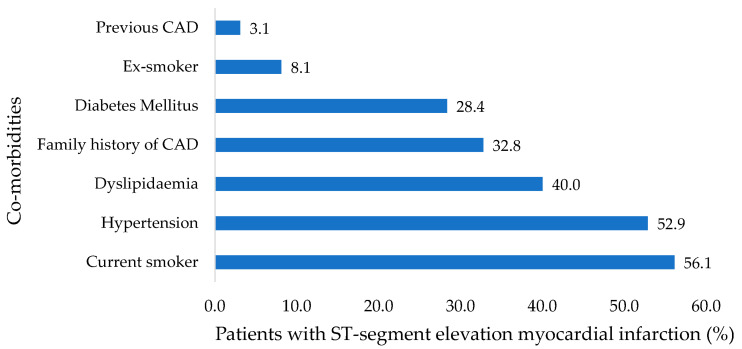
Co-morbidities for ST-segment elevation myocardial infarction. CAD: coronary artery disease.

The final study population comprised 677 patients, of which 533 (78.8%) were males. The mean age was 55.5 ± 11.3 years. Overall, 319 (47.1%) patients were Caucasians, 167 (24.7%) were Black, 143 (21.1%) were Indian or Asian, and 38 (5.6%) were of Mixed Ancestry. There were 380 (56.1%) patients with a history of smoking, 358 (52.9%) with hypertension, 271 (40.0%) with dyslipidemia, and 192 (28.4%) with diabetes mellitus (Figure 2). The median glycated haemoglobin (HbA1c) among diabetic patients was 8.7% (IQR: 7.3–10.5). There were 21 (3.1%) patients with a history of previous coronary artery diseases and 222 (32.8%) with a family history of heart disease. The rest of the baseline demographic and clinical characteristics are depicted in Table 1.

During the study period, 42 STEMI patients died, and the in-hospital all-cause mortality rate was 6.2% (95% CI: 4.5–8.3). Non-survivors were older, with a mean age of 60.5 ± 14.1 years, compared to survivors, with a mean age of 55.2 ± 11.1 years (*p* < 0.001). The median duration between the onset of chest pain to the catheterisation laboratory was shorter among non-survivors [1 (IQR: 0–2) vs. 2 (IQR: 1–3) days, *p* = 0.01]. During hospitalisation, non-survivors had lower blood pressures than survivors, with a mean systolic blood pressure of 104 vs. 126 mmHg (*p* < 0.001) and a mean diastolic blood pressure of 68 vs. 78 mmHg (*p* < 0.001). The baseline ECG heart rate was higher in non-survivors than survivors (99 vs. 83 beats per minute, *p* < 0.001).

### 3.2. Management Strategies

**Figure 3:**
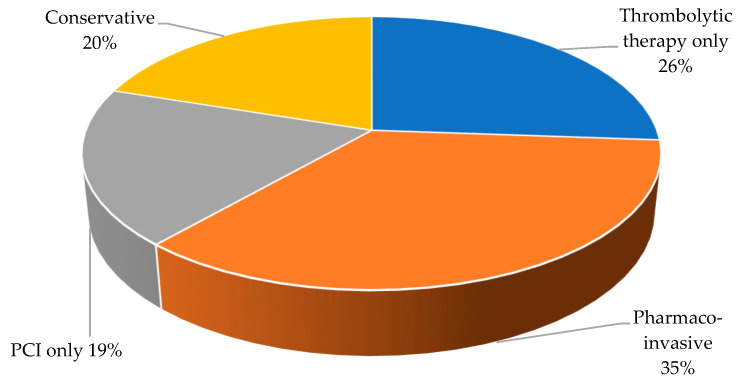
Management strategies in patients with ST-segment elevation myocardial infarction. PCI: percutaneous coronary intervention.

The pharmaco-invasive management strategy was implemented in 239 (35.3%) patients, 177 (26.1%) patients were treated with thrombolytic therapy only, 135 (19.9%) were managed conservatively, and 126 (18.6%) were treated with PCI only (Figure 3).

Among 239 STEMI patients managed with the pharmaco-invasive approach, 231 (96.6%) survived and 8 died (*p* = 0.027) (Table 2). Furthermore, on the univariable logistic regression analysis, the mortality risk was reduced in patients managed using the pharmaco-invasive approach (OR: 0.41; 95% CI: 0.19–0.90, *p* = 0.027). There was no association between the management strategy and mortality in the other treatment groups.

### 3.3. Coronary Angiography Findings, Complications and Predictors of In-Hospital Mortality

The left anterior descending artery (LAD) lesions were found in 78.6% of non-survivors. Triple-vessel disease was significantly associated with poor clinical outcomes, noted in 30.9% of non-survivors and 12.3% of survivors (*p* = 0.001) (Table 3). A total of 149 (22.0%) STEMI patients had PCI to the RCA; 102 (68.5%) of these patients were treated using the pharmaco-invasive approach and the rest of the patients (31.5%) received PCI only (*p* < 0.001).

In addition, 50.0% of the non-survivors required inotropes (*p* < 0.001), and a further 16.6% required cardiac pacing (*p* < 0.001). Among the 49 (7.2%) patients treated with dobutamine, 30 (61.2%) survived, and 19 patients died (*p* < 0.001). Twelve of the seventeen (70.6%) patients treated with adrenaline, and five of the six (83.3%) patients treated with phenylephrine did not survive (*p* < 0.001).

Post-intervention, 39 (5.8%) patients demonstrated haemodynamic instability, and 29 (74.3%) of these patients died (*p* < 0.001). Also, 11 (1.6%) patients had ventricular fibrillation, 10 (1.5%) had a ventricular tachycardia, and 9 (1.3%) had atrial fibrillation. ST-segment elevation myocardial infarction complicated by ventricular fibrillation and CHB were significantly associated with mortality (*p* < 0.001). The median duration of hospitalisation was three days (IQR: 2–6). The median time to death among non-survivors was 2.5 days (IQR: 1–9). Upon discharge from the hospital, 667 (98.5%) STEMI patients were prescribed oral angiotensin receptor blocker antagonists, 624 (92.2%) were on oral dual antiplatelet therapy, 517 (76.4%) were on beta-blockers, and 436 (64.4%) were on angiotensin-converting enzyme inhibitors. The prescription of mineralocorticoid receptor antagonists was uncommon, with only 67 (9.9%) patients on this oral medication.

In the age and sex-adjusted multivariable logistic regression model, the following predictors independently reduced the risk of in-hospital all-cause mortality: a higher estimated glomerular filtration rate (OR: 0.96; 95% CI: 0.93–0.98, *p* = 0.001), Killip class 1 (OR: 0.11; 95% CI: 0.03–0.36, *p* < 0.001), PCI to the RCA (OR: 0.03; 95% CI: 0.00–0.49, *p* = 0.015), and treatment using the pharmaco-invasive approach (OR: 0.16; 95% CI: 0.04–0.71, *p* = 0.015) (Table 4).

## 4. Discussion

In this study, we retrospectively reviewed the medical records of 677 patients diagnosed with STEMI. We found an in-hospital all-cause mortality rate of 6.2% (95% CI: 4.5–8.3). Ischemic heart disease, cerebrovascular disease, and hypertensive heart disease are SSA’s most common causes of cardiovascular morbidity and mortality [3]. Early studies showed a low incidence rate of IHD in SSA [9,10]. However, a steady rise was noted in the early 2000s [11,12]. The rising number of patients with IHD could be accounted for by rapid urbanisation, increased access to processed food and sedentary lifestyles. The previously reported lower incidence rate of IHD could be explained by underdiagnosis and paucity of research data reporting outcomes in STEMI patients residing in SSA, particularly those treated in state-owned hospitals. Limited access to diagnostic tools such as cardiac biomarkers reflecting myocardial injury, ECGs, and coronary angiography may result in the underdiagnosis of STEMI [13]. Furthermore, the incidence and prevalence rates of IHD reported in SSA in older research studies may be inaccurate, partly due to patients demising before arrival in hospitals equipped with catheterisation laboratories [14].

Smoking and hypertension were the most common risk factors in our study population, reported in 56.1% and 52.9% of STEMI patients, respectively. These were closely followed by dyslipidaemia in 40.0%, a family history of heart disease in 32.8%, diabetes mellitus in 28.4%, and obesity in 22.6%. The commonly reported modifiable risk factors for ASCVD include smoking, diabetes, hypertension, obesity, hyperlipidaemia, physical inactivity, and an unhealthy diet, which are becoming more prevalent in SSA. However, the true burden of risk factors for CAD has not been clearly defined due to fewer research studies performed in our region and deficient electronic health record systems for data collection [15]. The proportion of smokers residing in 29 SSA countries (South Africa not included in the analysis) is between 4.6 and 25.8% [3]. Hypertension is common in SA, with a higher % prevalence rate of 40%, reported in individuals residing in urban areas. In comparison, their counterparts in rural areas have a lower prevalence rate of 20% [16]. In 2014, diabetes mellitus had a prevalence rate of 7.1% among individuals of African descent [15]. In a meta-analysis of 117 studies with 294,063 participants, the pooled prevalence of dyslipidaemia in the general adult population residing in Africa was 25.5% (95% CI: 20.0–31.4) [17]. Guthold et al. surveyed 57,038 individuals from 22 African countries and found that at least 24.3% and 16.2% of females and males, respectively, did not meet the World Health Organisation’s physical activity recommendations [18]. The age-standardised prevalence of obesity among men and women 20 years and older in South Africa is 14.7% and 44.6%, respectively [19]. There is growing evidence that primary prevention of these risk factors in SSA may be a reasonable step to reduce the growing burden of ASCVD [20]. Other socio-economic factors identified as major psychosocial stressors contributing to an unhealthy diet, low physical activity, obesity, increased alcohol consumption, and smoking include poor housing, low income, poor sanitation, and limited access to healthcare facilities [21]. Also, most of the population in SSA resides in overpopulated informal settlements, an environment that is not optimal for establishing healthy behaviours.

Our study found an in-hospital all-cause mortality rate of 6.2% (95% CI: 4.5–8.3) among STEMI patients. The Kerala Acute Coronary Syndrome (ACS) Registry in India reported a mortality rate of 8.2% after evaluating 25,748 patients hospitalised with acute myocardial infarction, of which 9569 had STEMI [22]. Similarly, 18,631 patients in China with acute myocardial infarction, of which 13,815 were STEMI admissions, had a mortality rate of 7.0% [23]. Compared to other single-centre registries, our study demonstrated a lower mortality rate. For instance, Takagi et al. reported an in-hospital mortality rate of 9.2% in 2021 in a study consisting of 1735 participants [24], while Ali et al. found a mortality rate of 10% in their single-centre study involving 312 patients at Harzklinik Goslar in Germany [25].

The STEMI-related mortality in SSA is between 1.2 and 24.5% [2]. This wide range in the mortality rate is likely caused by the variation in the populations studied, where patients with different types of ACS presentations are included, such as STEMI, non-ST-segment elevation myocardial infarction (NSTEMI) and unstable angina. Also, limited access to coronary revascularisation may lead to a higher mortality rate. For instance, in a study by N’Guetta et al., the lowest mortality rate of 1.2% was observed [26]. Their study focused on patients hospitalised with ACS who underwent primary PCI. On the other end of the spectrum, the highest mortality rate of 27.4% observed in Ethiopia might have been influenced by the exceptionally long duration between the onset of ACS symptoms to presentation in the emergency department averaging approximately 92 h [27]. A review of the subset of data obtained from participants recruited in South Africa that are part of the ACCESS registry reported a STEMI mortality rate of 0.65% [4]. The nature of the study design may explain this very low mortality rate since the ACCESS registry was an observational study that enrolled an urban population at tertiary care facilities. Also, the enrolling centres were mainly private hospitals.

In 2017, South Africa was one of the five SSA countries with access to PCI, including Kenya, Côte d’Ivoire, Sudan, and Mauritania. South Africa had 62 PCI centres in 2017, most in private hospitals [28], compared to 12 catheterisation laboratories distributed over three SSA countries [29]. This discrepancy in access to catheterisation laboratories may explain the improved mortality outcomes in South Africa compared to other countries in the SSA region. Between 1990 and 2007, most low-and-middle-income countries (LMICs) focused on combatting the burden of communicable diseases [30]. Despite the growing burden of ASCVD, approximately 3% of global funding was allocated to non-communicable diseases in LMICs [30].

Atherosclerotic cardiovascular disease is traditionally associated with advanced age, particularly in high-income countries with higher life expectancy. In contrast, our study population had a mean age of 55 years. This finding is similar to reports from other regional studies [2,4]. Our study patients who died were older than those who survived, with a mean age of 60.5 years. While our population is younger than the rest of the world, the STEMI patients in our study that died were older in the context of life expectancy in our country.

The male gender was predominant, representing 79.2% of the population. The male gender is a non-modifiable risk factor for ASCVD. This is attributed to a higher burden of risk factors in men, with dyslipidaemia and smoking being the most prominent [31]. In addition, Larsson et al. suggested that the difference in body fat distribution could also account for the higher risk of ASCVD in men [32]. However, the Effect of Potentially Modifiable Risk Factors Associated with Myocardial Infarction (INTERHEART) case-control study found that in both males and females, abdominal obesity doubled the risk of acute myocardial infarction [33]. However, after adjusting for other risk factors, such as apolipoproteins, the risk of acute myocardial infarction was substantially reduced in both genders [33]. In our study, people of Caucasian ancestry comprised 47.1% of the study cohort, followed by those of African ancestry. In most LMICs, acute coronary syndromes are more prevalent among Caucasians [4]. It is worth noting that in South Africa, more than 80% of residents are of African descent [34]. Overall, our study findings align with the literature reporting a higher prevalence of IHD among Caucasian and Asian people.

Our study found that 59.5% of STEMI patients had culprit lesions in the LAD. However, the age and sex-adjusted multivariable logistic regression model showed that PCI targeting the RCA was protective and reduced the risk of in-hospital all-cause mortality. This finding suggests that successful complete revascularisation, including the RCA, may positively impact patient prognosis. Also, it emphasises the importance of accurately identifying the stenosed coronary artery and performing timely interventions to achieve optimal outcomes. However, it is also well established that the infarct size is usually smaller in patients with RCA lesions, which could explain these improved outcomes [35].

The United States Global Registry of Acute Coronary Events (GRACE) registry demonstrated that the heart rate is one of the eight predictors of mortality [36]. In our study, non-survivors had a mean heart rate of 99 ± 39.0 beats per minute (bpm). A heart rate between 70 and 79 bpm is considered low risk, while a rate above 80 bpm is associated with a 2.2–5.3-fold increased risk of in-hospital mortality [37]. Similarly, we found that an increase in the heart rate independently predicted mortality among STEMI patients. However, since the 95% confidence interval for the heart rate includes one, the interaction between the heart rate and mortality was not considered statistically significant, despite a *p*-value less than 0.05.

The eGFR emerged as a predictor of in-hospital mortality in our study population. This finding has also been supported by a study by McNamara et al. [38]. We found that non-survivors had an eGFR of less than 60 mL/min/1.73 m^2^. Impaired kidney function contributes to the deterioration of cardiovascular health, leading to worse outcomes in STEMI patients. Monitoring and managing eGFR becomes essential for risk stratification and improved patient care, aiming to reduce mortality. Additionally, the Killip class system is vital in stratifying individuals based on post-myocardial infarction heart failure severity. Our study supports existing literature by finding that patients classified as Killip class 1 had a favourable clinical outcome [39]. This observation is consistent with Killip class 1, representing patients with no cardiac failure and normal hemodynamic status, thereby explaining the better survival rates observed in these patients. By considering both the eGFR and Killip classification in STEMI patients, clinicians can gain valuable insights into the severity of the condition and tailor appropriate management strategies. These findings reinforce the significance of kidney function assessment and the Killip classification as essential tools in predicting outcomes and optimising patient care in STEMI patients.

Troponin release follows predictable kinetics, and peak levels correlate with ischemic time and the amount of at-risk myocardium [40]. Additionally, troponins exhibit excellent cardiac specificity, and higher peak troponin levels have been associated with ventricular dysfunction and mortality [41,42]. In our study, non-survivors had higher levels of troponin, almost twice as high as that of survivors. However, troponin levels did not emerge as a significant predictor of mortality in the multivariable logistic regression model. Hyperglycaemia has also been reported as a significant predictor of mortality [43]. However, our study did not document nor analyse blood glucose levels among STEMI patients. Although fingerstick testing is routinely performed in patients admitted to our hospital, formal random glucose testing is rarely performed due to resource constraints unless there is a specific clinical indication. We were, however, able to assess glucose control using HbA1c levels in diabetic patients.

Minimising prehospital delays and implementing a primary PCI strategy in high-income countries has significantly decreased morbidity and mortality associated with STEMI [1]. In our study population, the median duration between the onset of chest pain and catheterisation was approximately two days. Among the participants who underwent coronary angiography, 53.9% received either primary or ad hoc PCI, with or without thrombolytic therapy. It is worth noting that a minority of patients received true primary PCI, attaining <2 h from first medical contact to “balloon” time. Resource constraints, an overwhelmed referral network, and potentially suboptimal triage systems have led to the standard of care being a pharmaco-invasive strategy, where patients are often treated with thrombolytic therapy before catheterisation. This approach was followed in our clinical setting, with 35.3% of patients undergoing this coronary revascularisation strategy. Our study reported a prolonged pain-to-balloon time but still reported a reasonable survival rate, likely attributed to our institution being a tertiary referral centre. This characteristic of our centre may have led to the inclusion of patients who survived until referral. At the same time, those who died before reaching our institution were not part of the study’s final analysis.

While our data do not emphasise the importance of timely catheterisation, as the mortality was higher in those who received earlier PCI, it speaks to the value of the pharmaco-invasive approach employed within our region. A plausible explanation for the higher mortality in the patients that received earlier PCI is that they may have been more critically ill and were therefore prioritised for catheterisation, and due to the nature of their presentation and were, therefore, more likely to die. In SSA, thrombolytic therapy is often readily available, and some literature has shown a similar clinical benefit to primary PCI [44]. Due to its lower cost, Streptokinase is the most accessible thrombolytic agent in SSA [2,4], although Alteplase is also used in South Africa [45]. In our study population, 61.4% received thrombolytic therapy. We hypothesise that the inadequate thrombolysis rates may have resulted from limited access to thrombolytics, late presentation beyond the therapeutic time window for thrombolytic therapy, and physician inertia.

The pharmaco-invasive approach holds paramount importance for STEMI patients in SSA. Given the challenges in accessing timely primary PCI, this strategy combines pharmacological treatment with early fibrinolysis and subsequent transfer for PCI, reducing ischemic time and improving outcomes. Implementing this approach in the region’s healthcare systems may significantly improve STEMI management, save lives, and alleviate the burden of cardiovascular diseases. However, concerted efforts from healthcare stakeholders, policymakers, and funding agencies are crucial to maximise the impact of the pharmaco-invasive approach on patient outcomes. Improving access to catheterisation laboratories will also require the retention of cardiologists in state-owned facilities. In South Africa, the private sector enjoys an increasing number of cardiologists, while there are fewer cardiologists in state-owned facilities.

The study had several limitations. The retrospective nature of the study design introduced numerous challenges, such as illegible documentation of clinical data and incomplete medical records. The study data were obtained from a single centre, limiting the generalisability of the findings. Electrocardiogram parameters were mainly gathered from clinical notes made by the cardiologists, and a few patients had legible ECG printouts. The definitive cause of death was not routinely documented. Also, there was an overlap with the use of the third and fourth universal definitions of myocardial infarction, resulting in the third definition from 2015 to 2018 and the fourth definition from 2018 to 2019. Due to the retrospective nature of the study design, we have presented the timelines from first medical contact to “balloon time” in calendar days. We were unable to determine the intervention timelines in hours. Furthermore, pertinent clinical parameters such as the left ventricular ejection fraction and serum glucose levels were not collected and analysed as part of the study. Despite these limitations, our study provides valuable insights into the burden of co-morbidities in STEMI patients and estimated the rate of in-hospital all-cause mortality in STEMI patients managed in a tertiary academic centre in Johannesburg, South Africa.

## 5. Conclusions

In our study, the in-hospital, all-cause mortality rate among patients with STEMI was 6.2%. This mortality rate is comparable to that published in high-income and other LMICs. A pharmaco-invasive approach significantly reduced the risk of in-hospital all-cause mortality. In SSA, large prospective multicentre observational registries are still required to accurately estimate the mortality rate and define STEMI mortality predictors in our region.

## Figures and Tables

**Table 1:** Baseline demographic and clinical parameters in patients with ST-segment elevation myocardial infarction at the time of hospitalisation.

Variables	All	All-Cause Mortality		
	Patients	No	Yes	
	*n* = 677	(*n* = 635) (93.8%)	(*n* = 42) (6.2%)	*p*-Value
Age, years	55.54 ± 11.39	55.21 ± 11.11	60.54 ± 14.11	<0.001
Male	533 (78.73)	503 (79.21)	30 (71.43)	0.233
Co-morbidities				
Hypertension	358 (52.88)	335 (52.76)	23 (54.76)	0.801
Diabetes Mellitus	192 (28.36)	179 (28.19)	13 (30.95)	0.700
Dyslipidaemia	271 (40.03)	255 (40.16)	16 (38.10)	0.792
Obesity	153 (22.60)	144 (22.68)	9 (21.43)	0.851
Chronic kidney disease	17 (2.51)	17 (2.68)	0 (0.00)	0.283
Previous CAD	21 (3.10)	20 (3.15)	1 (2.38)	0.781
Smoking				
Current smoker	380 (56.13)	362 (57.01)	18 (42.86)	0.073
Ex-smoker	55 (8.12)	49 (7.72)	6 (14.29)	0.131
Vital signs				
Systolic BP (mmHg)	124.41 ± 26.15	125.77 ± 25.09	103.46 ± 32.94	<0.001
Diastolic BP (mmHg)	78.65 ± 18.49	79.36 ± 18.12	67.76 ± 20.92	<0.001
Heart rate	84.38 ± 22.26	83.42 ± 20.37	98.83 ± 39.06	<0.001
NYHA class				
1	601 (88.77)	583 (91.81)	18 (42.86)	<0.001
2	37 (5.47)	30 (4.72)	7 (16.67)	0.001
3	17 (2.51)	14 (2.20)	3 (7.14)	0.048
4	22 (3.25)	8 (1.26)	14 (33.33)	<0.001
Killip class				
1	576 (85.08)	560 (88.19)	16 (38.10)	<0.001
2	30 (4.43)	26 (4.09)	4 (9.52)	0.098
3	24 (3.55)	17 (2.68)	7 (16.67)	<0.001
4	47 (6.94)	32 (5.04)	15 (35.71)	<0.001
Biochemical Parameters				
Troponin T (ng/L)	3556 (1495–7189)	3444 (1389–6893)	6774 (2732–10,000)	0.004
CK MB mass (µg/L)	82.67 (20.17–239.1)	72.35 (19.38–203.6)	268 (207.2–268)	0.013
Haemoglobin (g/dL)	14.34 ± 2.20	14.34 ± 2.18	14.32 ± 2.61	0.972
Sodium (mmol/L)	138 ± 9.12	139.21 ± 7.87	135.29 ± 19.80	0.007
Potassium (mmol/L)	4.2 (3.9–4.6)	4.2 (3.9–4.6)	4.45 (4.0–5.2)	0.021
Urea (mmol/L)	5.6 (4.4–7.4)	5.5 (4.4–7.1)	7.9 (6.0–11.7)	<0.001
Creatinine (µmol/L)	87 (73–107)	86 (72.5–104)	115 (82–149)	<0.001
eGFR (mL/min/1.73 m^2^)	78.35 ± 27.21	79.78 ± 26.52	56.64 ± 28.62	<0.001
Electrocardiogram findings				
Sinus rhythm	610 (90.10)	581 (91.50)	29 (69.05)	<0.001
Atrial fibrillation	12 (1.77)	10 (1.57)	2 (4.76)	0.130
Atrial flutter	3 (0.44)	3 (0.47)	0 (0.00)	0.655
Ventricular tachycardia	7 (1.03)	6 (0.94)	1 (2.38)	0.373
Ventricular fibrillation	5 (0.74)	3 (0.47)	2 (4.76)	0.002
Complete heart block	22 (3.25)	16 (2.52)	6 (14.29)	<0.001
Left bundle branch block	19 (2.81)	18 (2.83)	1 (2.38)	0.186
Right bundle branch block	24 (3.55)	20 (3.15)	4 (9.52)	0.186

BP: blood pressure, CAD: coronary artery disease, CK: creatine kinase, eGFR: estimated glomerular filtration rate, NYHA: New York Heart Association.

**Table 2:** Management strategies and mortality outcomes in patients with ST-segment elevation myocardial infarction.

Management Strategy	All-Cause Mortality		*p*-Value
	No (*n* = 635) (93.8%)	Yes (*n* = 42) (6.2%)	
Pharmaco-invasive	231(36.4)	8 (19.0)	0.023
Thrombolytic therapy only	166 (26.1)	11 (26.2)	0.994
Conservative management	122 (19.2)	13 (30.9)	0.065
Percutaneous coronary intervention only *	116 (18.3)	10 (23.8)	0.371

* Refers to primary or ad hoc percutaneous coronary intervention without prior thrombolysis.

**Table 3:** Coronary angiography findings and percutaneous coronary interventions in patients with ST-segment elevation myocardial infarction.

Variables	All	All-Cause Mortality		
	Patients	No	Yes	
	*n* = 677	(*n* = 635) (93.8%)	(*n* = 42) (6.2%)	*p*-Value
Vessels involved				
Right coronary artery	351 (51.85)	330 (51.97)	21 (50.00)	0.850
Left main coronary artery	14 (2.07)	12 (1.89)	2 (4.76)	0.205
Left circumflex artery	156 (23.04)	142 (22.36)	14 (33.33)	0.102
Left anterior descending artery	403 (59.53)	370 (58.27)	33 (78.57)	0.009
Obtuse marginal/Ramus	29 (4.28)	27 (4.25)	2 (4.76)	0.874
First diagonal branch	13 (1.92)	12 (1.89)	1 (2.38)	0.822
Distribution of disease on angiography				
Single vessel disease	390 (57.61)	369 (58.11)	21 (50.00)	0.303
Double vessel disease	143 (21.12)	137 (21.57)	6 (14.29)	0.262
Triple vessel disease	91 (13.44)	78 (12.28)	13 (30.95)	0.001
No lesion *	20 (2.95)	20 (3.15)	0 (0.00)	0.243
Percutaneous coronary intervention				
Percutaneous coronary intervention	365 (53.91)	347 (54.65)	18 (42.86)	0.125
Left main coronary artery	1 (0.15)	1 (0.16)	0 (0.00)	0.797
Left anterior descending artery	187 (27.62)	172 (27.09)	15 (35.71)	0.226
Left circumflex artery	40 (5.91)	36 (5.67)	4 (9.52)	0.305
Right coronary artery	149 (22.01)	146 (22.99)	3 (7.14)	0.016
Obtuse marginal artery	1 (0.15)	1 (0.16)	0 (0.00)	0.797
Obtuse marginal/Ramus	8 (1.18)	8 (1.26)	0 (0.00)	0.464

* No lesion: refers to resorbed thrombus, lysed thrombus, and myocardial infarction with non-obstructive coronary arteries (MINOCAs).

**Table 4:** Univariable and multivariable logistic regression analysis of predictors of in-hospital all-cause mortality in patients with ST-segment elevation myocardial infarction.

Variable	Univariable Logistic Regression		Multivariable Logistic Regression			
	Unadjusted OR (95% CI)	*p*-Value	Unadjusted OR (95% CI)	*p*-Value	Adjusted OR * (95% CI)	*p*-Value
Age	1.04 (1.01–1.07)	0.004			1.04 (1.00–1.08)	0.082
Duration between the onset of chest pain and catheterisation (days)	0.88 (0.75–1.03)	0.112				
Systolic BP (mmHg)	0.96 (0.95–0.98)	<0.001	0.97 (0.94–1.01)	0.117	0.97 (0.93–1.00)	0.075
Diastolic BP (mmHg)	0.96 (0.94–0.98)	<0.001	1.02 (0.96–1.07)	0.527	1.03 (0.97–1.08)	0.344
Troponin (ng/L)	1.00 (1.00–1.00)	0.007	1.00 (1.00–1.00)	0.838	1.00 (1.00–100)	0.756
Sodium (mmol/L)	0.98 (0.96–1.00)	0.030	0.99 (0.95–1.02)	0.414	0.99 (0.95–1.02)	0.463
Potassium (mmol/L)	1.03 (1.00–1.06)	0.038	1.03 (0.98–1.08)	0.211	1.03 (0.98–1.09)	0.268
Urea (mmol/L)	1.04 (1.00–1.07)	0.014	1.03 (0.98–1.08)	0.196	1.03 (0.98–1.08)	0.228
eGFR (mL/min/1.73 m^2^)	0.96 (0.95–0.98)	<0.001	0.96 (0.93–0.98)	<0.001	0.96 (0.93–0.98)	0.001
NYHA class 1	0.07 (0.03–0.13)	<0.001				
NYHA class 2	4.03 (1.66–9.83)	0.002				
NYHA class 3	3.41 (0.94–12.37)	0.062				
Killip class 1	0.08 (0.04–0.16)	<0.001	0.13 (0.04–0.39)	<0.001	0.11 (0.03–0.36)	<0.001
Killip class 2	2.47 (0.82–7.43)	0.109				
Sinus rhythm	0.21 (0.10–0.42)	<0.001				
ECG Heart Rate (bpm)	1.02 (1.01–1.04)	<0.001	1.02 (1.00–1.04)	0.022	1.02 (1.00–1.04)	0.022
Anterior MI	1.73 (0.90–3.31)	0.099				
PCI to the RCA	0.26 (0.08–0.85)	0.025	0.04 (0.00–0.53)	0.015	0.03 (0.00–0.49)	0.015
Pharmaco-invasive approach	0.43 (0.20–0.96)	0.038	0.20 (0.05–0.79)	0.022	0.16 (0.04–0.71)	0.015

BP: blood pressure, CI: confidence interval, ECG: electrocardiogram, eGFR: estimated glomerular filtration rate, MI: myocardial infarction, NYHA: New York Heart Association, OR: odds ratio, PCI: Percutaneous coronary intervention, RCA: Right coronary artery. * Age and sex-adjusted odds ratio.

## Data Availability

The dataset used in this study can be made available on request.

## Supplementary Materials

The following supporting information can be downloaded at: https://www.mdpi.com/article/10.3390/jcdd10080348/s1, Univariable and multivariable logistic regression models predicting in-hospital all-cause mortality in patients with ST-segment elevation myocardial infarction.
