# Supplementary material for: In-Hospital Mortality Outcomes of ST-Segment Elevation Myocardial Infarction: A Cross-Sectional Study from a Tertiary Academic Hospital in Johannesburg, South Africa
Source: J Cardiovasc Dev Dis. 2023 Aug 15;10(8):348. doi: 10.3390/jcdd10080348 (PMC10455389; doi:10.3390/jcdd10080348)
Supplement: Supplementary file 1 [file jcdd-10-00348-s001.zip › jcdd-2536489-supplementary.pdf]

Supplementary file

UNIVARIABLE LOGISTIC REGRESSION MODEL (NO ADJUSTMENT FOR CONFOUNDERS)

Variables with a p-value < 0.1 on the Pearson's chi-square test, Wilcoxon ranksum test or Students' t-test:

logit mortality age, or nolog

logit mortality chestpain_cath,or nolog

logit mortality systolic,or nolog

logit mortality diastolic, or nolog

logit mortality troponin,or nolog

logit mortality ck_mb,or nolog

logit mortality sodium,or nolog

logit mortality potassium,or nolog

logit mortality urea,or nolog

logit mortality creatinine ,or nolog

logit mortality egfr,or nolog

logit mortality i.nyha1,or nolog

logit mortality i.nyha_class2,or nolog

logit mortality i.nyha_class3,or nolog

logit mortality i.nyha_class4,or nolog

logit mortality i.killip_class1,or nolog

logit mortality i.killip_class2,or nolog

logit mortality i.killip_class3,or nolog

logit mortality i.killip_class4,or nolog

logit mortality i.sinus,or nolog

logit mortality i.ventrfib ,or nolog

logit mortality i.heartblock ,or nolog

logit mortality ecg_rate,or nolog

logit mortality i.anterior_MI ,or nolog

logit mortality i.nolesion,or nolog

logit mortality i.dobutamine,or nolog

logit mortality i.adrenaline,or nolog

logit mortality i.phenylephrine ,or nolog

logit mortality i.pacing ,or nolog

logit mortality i.pci_rca, or nolog

logit mortality i.haemodynamic_instability ,or nolog

logit mortality i.Group1_pharmaco_invasive ,or nolog

The following variables were omitted in the multivariable model due to collinearity. These variables were identified based on a inflated odds ratio, high standard error or wide confidence interval.

1. Inotropes
2. Creatinine
3. Complete heart block
4. Ventricular fibrillation
5. Sinus rhythm
6. NYHA class 4
7. Killip class 3
8. Killip class 4
9. Phenylephrine
10. Haemodynamic_instability
11. Dobutamine
12. Adrenaline
13. Pacing
14. Anterior myocardial infarction
15. No lesion (normal coronary angiography)

The following variables were removed by Stata, the statistical analysis software:

1. CKMB: "ck_mb > 211.3 predicts data perfectly"
2. NYHA class 1: "predicts death perfectly"

FINAL MULTIVARIABLE REGRESSION MODEL

1. Unadjusted (crude odds ratio):

logistic mortality systolic diastolic troponin sodium potassium urea egfr   i.killip_class1 ecg_rate i.pci_rca i.Group1_pharmaco_invasive

2. Adjusted for age and sex:

```
logistic mortality age sex systolic diastolic troponin  sodium potassium urea  egfr   i.killip_class1
ecg_rate    i.pci_rca  i.Group1_pharmaco_invasive
```
